# Are Polymorphisms in Genes Relevant to Drug Disposition Predictors of Susceptibility to Drug-Induced Liver Injury?

**DOI:** 10.1007/s11095-016-2091-1

**Published:** 2016-12-27

**Authors:** Ann K. Daly

**Affiliations:** Institute of Cellular Medicine, Medical School, Newcastle University, Framlington Place, Newcastle upon Tyne, NE2 4HH UK

**Keywords:** ABC transporter, cytochrome P450, drug-induced liver injury, UDP-glucurononosyltransferase

## Abstract

Despite considerable progress in identifying specific HLA alleles as genetic risk factors for some forms of drug-induced liver injury, progress in understanding whether genetic polymorphisms relevant to drug disposition also contribute to risk for developing this serious toxicity has been more limited. Evidence from both candidate-gene case control studies and genome-wide association studies is now discussed. In the case of genes relevant to drug metabolism, polymorphisms in cytochromes P450, UDP-glucuronosyltransferases, N-acetyltransferases and glutathione S-transferases as risk factors for DILI are assessed. The relevance of ABC transporters to drug-induced liver injury is also considered, together with data showing associations of particular ABCB11, ABCB1 and ABCC2 polymorphisms with some forms of drug-induced liver injury. Very few of the associations with genes relevant to drug disposition that have been reported have been well replicated. Even apparently well-studied associations such as that between isoniazid liver injury and N-acetyltransferase 2 slow acetylators remain problematic, though it seems likely that polymorphisms in drug metabolism genes do contribute to risk for some specific drugs. A better understanding of genetic risk factors for drug-induced liver injury will require further genome-wide association studies with larger numbers of cases, especially for forms of drug-induced liver injury where HLA genotype does not appear to be a risk factor.

## Introduction

Drug-induced liver injury (DILI) is one of the most common reasons for drug withdrawal, either at a late stage of the drug development process or soon after licensing (1). It is also an important contributor to serious liver disease, though the majority of DILI cases that lead to acute liver failure are due to acetaminophen overdose rather than the rarer idiosyncratic DILI where the disease develops after the causative drug is used at the recommended dose (2). It is generally considered that metabolism of the causative drug to a reactive intermediate is an important step in the DILI process (3). In addition, impaired membrane transport in the hepatocyte, affecting either drug transport directly or bile acid transport, is considered to be a contributor to DILI, possibly by promoting accumulation of reactive intermediates or of toxic bile acids in hepatocytes (4). There is extensive data available on the functional significance of a range of polymorphisms in the genes encoding drug metabolizing enzymes and transporters. This has enabled studies on the possibility of a relationship between genotype for biologically plausible polymorphisms in these genes and risk of DILI development to be performed, resulting in some interesting observations giving insights into the underlying mechanism, though this overall area is still unclear with an absence of strong effects reported. Most genetic associations reported to date for DILI relate to HLA genes, pointing to an important role for an inappropriate immune response (5). However, not all forms of DILI show an association with a particular HLA genotype. The formation of reactive intermediates may also contribute to an inappropriate immune response so a role for genes contributing to drug disposition alongside HLA genes in DILI remains plausible.

### Cytochromes P450

Because of the ability of the cytochromes P450 to metabolize a wide range of drugs to reactive intermediates and also the extent of interindividual variation in levels of P450 enzymes, polymorphisms in these genes are good candidates as susceptibility factors for DILI. An important role for P450 genes in DILI susceptibility has not been confirmed to date, though there are a few reports of associations in relation to particular drugs. In the concentration-dependent form of DILI seen in acetaminophen overdose, activation to the N-acetyl-p-benzoquinoneimine (NAPQI) reactive metabolite is the well established mechanism for toxicity. A recent study has shown a strong correlation between levels of total P450-generated metabolites and serious liver toxicity (6). CYP2E1, CYP1A2 and CYP3A are well established contributors to NAPQI production (7). Studies on the relationship between CYP genotype and development of DILI following overdose are very limited but one relatively large study reported a borderline significant relationship between CYP3A5*1 in patients who took an intentional overdose and developed acute liver failure (8). This allele is associated with CYP3A5 expression and therefore, on average, an overall higher CYP3A activity. The finding is biologically plausible but needs confirmation in another similar cohort.

Findings on relationship between CYP alleles and idiosyncratic DILI are quite limited. There is a report from the 1980s that CYP2D6 poor metabolizers were more susceptible to perhexiline-induced DILI but, since this drug was withdrawn soon after the study, follow-up was not possible (9). More recently, a role for another P450 isoform, CYP2B6, in two different forms of idiosyncratic DILI has been suggested. For DILI due to ticlopidine, an upstream SNP (rs7254579 which results in -2320T>C) which has been reported to be associated with increased CYP2B6 expression (10) was significantly more common among cases (11). It is possible that this variant could be associated with increased reactive intermediate levels leading to increased risk of DILI. On the other hand, in cases of DILI due to efavirenz, the CYP2B6*6 allele appears to be more common (12). This variant is associated with lower than normal CYP2B6 activity suggesting that for efavirenz, accumulation of the parent drug might be an important factor in DILI.

A possible association between CYP2E1 and DILI due to isoniazid has been fairly extensively investigated but studies have not shown consistent associations. This may reflect limited effects of common CYP2E1 genetic polymorphisms on enzyme activity and expression and this is still a confusing area. Most studies concerned the CYP2E1*5 variant (rs2031920) (an allele often detected using the restriction enzyme *Rsa*I) for which the biological significance remains unclear (for review see (13)). More detailed studies investigating other CYP2E1 polymorphisms for this form of DILI might be worthwhile, though a genome-wide association study (GWAS) involving 28 cases of isoniazid DILI found no evidence for a contribution from any CYP2E1 polymorphisms (or other genes) to susceptibility (14).

### UDP-glucuronosyltransferases (UGT)

Members of the UGT family make probably the largest contribution to phase II metabolism of drugs implicated in DILI. They are relevant as DILI risk factors because of their role in detoxicating reactive metabolites (or the parent drug) but also because acylglucuronides generate by glucuronidation of carboxyl groups are reactive intermediates which may themselves bind covalently to proteins triggering direct toxicity or an inappropriate immune response (15). The main associations involving UGT genes reported to date for DILI concern acetaminophen overdose and idiosyncratic reactions induced by diclofenac and tolcapone.

A number of different UGT isoforms contribute to acetaminophen glucuronidation, including 3 members of the UGT1A family which all originate from the same UGT1A gene. Studies with a human liver bank indicated that three polymorphisms at the 3’-untranslated region correlate with an increased acetaminophen glucuronidation activity, possibly due to effects on exon splicing. Carriage of one of these polymorphisms (rs8330) appeared to protect against the development of acute liver failure in patients who had suffered an unintentional overdose (16).

Tolcapone, an inhibitor of catechol-O-methyltransferase used in treatment of Parkinson’s disease, can give rise to elevated transaminase levels in some patients. Several polymorphisms in the glucuronidating enzyme UGT1A6 promoter region were found to be significantly associated with elevated transaminase levels (17). Because these polymorphisms appear to be associated with decreased enzyme levels, it was suggested that the toxicity might be linked to slow metabolism of the parent drug. It was suggested subsequently that promoter region polymorphisms in the nearby UGT1A9 may also be relevant to DILI from this drug (18).

Diclofenac is a widely prescribed non-steroidal antiinflammatory which is a relatively common cause of DILI worldwide. This compound undergoes glucuronidation by UGT2B7. In a study on susceptibility to diclofenac-related DILI, possession of UGT2B7*2, which is believed to be associated with higher than normal glucuronidating activity, was associated with an increased risk of toxicity (19). This suggests that the underlying mechanism for toxicity might involve production of high levels of diclofenac acylglucuronide. A slightly larger follow-up study (*n* = 30) which included some of the samples from the earlier diclofenac DILI study involved a genome-wide association study approach. In a substudy of genes relevant to drug disposition only, SNPs in the UGT2B region which were in linkage disequilibrium with the UGT2B7*2-related SNPs were more common in the diclofenac DILI cases (14).

### N-acetyltransferase 2

The phase II enzyme N-acetyltransferase 2 (NAT2) has only a minor role in drug metabolism generally but a key role in metabolism of isoniazid, an important drug in the treatment of tuberculosis (TB), which is usually used in combination with other drugs. NAT2 is subject to a well studied genetic polymorphism with approx. 50% of individuals from a number of different ethnic groups lacking this enzyme activity entirely, usually due to the presence of a coding region polymorphism which affects enzyme stability or activity. Isoniazid is a common cause of DILI. Acetylhydrazine, an isoniazid metabolite which can undergo metabolism by cytochrome P450 to a toxic metabolite or by NAT2 to the less toxic diacetylhydrazine, may be the cause of toxicity but the parent drug, which is seen at higher plasma levels in those with no NAT2 activity, may also contribute (20). A possible association between NAT2 genotype and DILI susceptibility has been investigated extensively. As reviewed recently, there are a large number of studies on this aspect with the majority suggesting that absence of NAT2 activity (slow acetylator phenotype) is a risk factor for isoniazid DILI (21). However, the GWAS discussed above involving 28 cases of isoniazid DILI did not detect a signal at the *NAT2* gene (14). Though reports showing this association continue to appear (22–24), there are limitations to most since the studies generally involve very small numbers of cases and often concern cases with very mild DILI which is quite common but usually resolves without the need to discontinue drug treatment. An added complication is that patients are usually also prescribed pyrazinamide, which can also cause DILI but is not metabolized by NAT2.

### Glutathione S-transferases

The glutathione S-transferases (GST) metabolize a range of drugs by glutathione conjugation and also have a role in protection against oxidative stress. Two GST isoforms, GSTM1 and T1, are absent in significant numbers of individuals due to large gene deletions. Candidate gene studies on GST in relation to a number of types of DILI have been performed. These suggest that individuals homozygous null for both *GSTM1* and *GSTT1* are at increased risk of DILI due to troglitazone, amoxicillin-clavulanate and also possibly a wider range of drugs (25–27). Data for isoniazid-related DILI is rather contradictory with two studies reporting an increased frequency of *GSTM1* homozygous null genotypes only (28,29) and a third increased *GSTT1* homozygous null only (30). The finding on troglitazone is consistent with the finding that GSTM1 but not GSTT1 has a role in detoxication of a quinone metabolite of this drug (31) but there appears to be no direct role for GST in amoxicillin-clavulanate or isoniazid elimination. Independent replication of these suggested GST associations is still needed.

### ABC Transporters

ABC transporters, including the specific bile acid transporter BSEP which is encoded by *ABCB11*, are of considerable interest in relation to DILI, especially for cholestatic forms of the disease where bile acids accumulate within hepatocytes, resulting in local toxicity. Other ABC transporters, including MDR1 and MRP2 which both contribute to biliary excretion of a range of drugs, are also plausible candidates as DILI risk factors (4). In addition to genetic variation, inhibition of the normal ABC transporter function by certain drugs may contribute to risk of DILI (32). It is possible that inhibition may be more problematic in individuals where transporter expression or activity is lower than normal due to the presence of genetic polymorphisms. In general, genome-wide association studies on DILI have failed to demonstrate any significant signals from ABC transporters, suggesting that any role as a genetic risk factor is relatively small (compared with, for example, HLA genotype) or is specific to only a few specific drugs (14). Some slightly more positive findings have emerged from candidate gene studies.

In a study on a small group of patients who had suffered DILI relating to a range of different drugs, an association between cholestatic injury and a polymorphism in exon 13 of *ABCB11* was reported (33). The effect on disease risk from this common polymorphism was small and it therefore seems unlikely to be a major risk factor for DILI. In a more detailed study involving genotyping for a larger number of polymorphisms including the exon 13 variant, no association with DILI was detected (34). In contrast to the original finding about *ABCB11* and cholestatic DILI, a further study on the exon 13 variant suggested that possession of this allele was associated with increased risk of hepatocellular disease, especially with non-steroidal antiinflammatory drugs (35).

MRP2 has a major role in the biliary excretion of many glucuronide conjugates and is encoded by *ABCC2*. In the study on diclofenac DILI described above where an association with UGT2B7 polymorphisms was detected, carriage of an upstream polymorphism in *ABCC2* (rs717620; −24C>T) was found to be significantly more common among DILI cases (19). This finding is consistent with increased levels of the reactive diclofenac acyl glucuronide being linked to toxicity on the basis of findings that −24C>T results in lower expression of the MRP2 protein which would favor cellular accumulation of the glucuronide (36). In a Korean study on DILI caused by a range of drugs, a polymorphism in linkage disequilibrium with −24C>T was a significant risk factor for the development of hepatocellular toxicity whereas a second promoter region polymorphism was a risk factor for cholestatic or mixed disease (37). These findings regarding *ABCC2* −24C>T are in broad agreement with the data on diclofenac toxicity. In a substudy concerned only with drug disposition genes which formed part of a larger GWAS on DILI, the lowest p values for drug disposition-related genes were seen for a series of *ABCC2* polymorphisms (14). More recently, a study based in Spain on ABC transporter polymorphisms as risk factors for DILI found some associations with a rare *ABCC2* genotype and also reported an association between an extended *ABCC2* haplotype and disease severity (38).

Genotypes for another transporter gene, *ABCB1* which codes for MDR1, have been studied in patients with DILI due to nevirapine. In African DILI cases, there was a significantly decreased frequency of the *ABCB1* SNP 3435C>T (rs1045642) (39) and a similar association was also reported in a US patient group (40). However, a subsequent study on a separate group of European nevirapine DILI patients failed to confirm this association (41). No association between *ABCB1* polymorphisms and DILI more generally has been found to date.

Two other ABC transporters, MRP3 and MRP4 (encoded by the *ABCC3* and *ABCC4* genes) are found on the basolateral membrane of the hepatocyte and are also of interest in relation to DILI susceptibility when data from model systems is considered. MRP4 is expressed at higher levels than MRP3 in human hepatocytes and also appears more important in efflux of bile acids, especially in cholestasis (42,43). It has been suggested that MRP4 inhibition by some compounds is an important underlying mechanism for bile acid accumulation in cholestatic DILI (44). For example, troglitazone and its sulfate metabolite inhibit both BSEP and MRP4 (45). In the case of MRP3, inhibition of transport by drugs appears less relevant to DILI generally but there is evidence that in a Mrp3 mouse knockout model, basolateral efflux of diclofenac acylglucuronide is lost and that the animals show increased gastrointestinal ulceration (46). It is possible that hepatic accumulation of diclofenac acylglucuronide when both MRP2 and MRP3 levels are low due to genetics or drug inhibition could be a factor in the human liver toxicity (47). However, as with the other candidate transporter polymorphisms, to date no association involving *ABCC3* (MRP3) has been detected in a GWAS (14).

## Concluding Remarks and Future Perspectives

There are essentially no well-replicated and completely convincing data showing associations between DILI susceptibility and specific genes relevant to drug disposition. Table I lists selected examples from those discussed in detail above which show reasonable biological plausibility but where further confirmation is still needed. The availability of GWAS approaches has added to the complexity since essentially no previously reported associations (e.g. *NAT2* genotype and isoniazid DILI) have been replicated in GWAS up to now. GWAS on DILI has been successful in demonstrating HLA associations with odds ratios of approx. 2.5 or higher, even when case numbers are small, but generally other risk factors, which may well include some of the genes relevant to drug disposition discussed above, have not yet emerged from these analyses. The failure to detect these additional risk factors is not necessarily that they don’t exist but possibly because the effect sizes reported in small candidate gene studies are biased towards only strongly positive findings being reported. A better understanding of genetic risk factors for these rare events will require larger numbers of well phenotyped cases to be collected and analyzed using GWAS and DNA sequencing approaches. This is feasible through international collaboration for drugs in current use. However, where drugs have been withdrawn from the market due to serious DILI, the number of cases available for genetic studies may be relatively small and access to them difficult. The need for larger studies applies to the more common concentration-dependent toxicity seen in acetaminophen overdose as well as to idiosyncratic DILI.

**Table I:** Selected Associations Between DILI Susceptibility and Genes Relevant to Drug Disposition with Biological Plausibility

Drug	Gene and allele	Comments	Reference
Acetaminophen	CYP3A5*1	Intentional overdose only. Confirmation needed	(8)
Acetaminophen	UGT1A4 rs8330 (2042C>G)	Unintentional overdose only. Confirmation needed	(16)
Diclofenac	UGT2B7*2	Candidate gene finding partly confirmed in additional cases	(14,19)
Diclofenac	ABCC2 rs717620 (−24C>T)	Confirmation needed	(19)
Efavirenz	CYP2B6*6	Confirmation needed	(12)
Isoniazid	NAT2 slow acetylator alleles	Association is based on small studies which generally involve a mild phenotype. Not yet confirmed by GWAS.	(14,21)
Ticlopidine	CYP2B6 rs7254579 (−2320T>C)	Confirmation needed	(11)
Tolcapone	UGT1A6/1A9 (various SNPs)	Confirmation needed	(17,18)

Where the increased risk of an ADR such as DILI due to a particular genotype is in the order of 1 to 2 fold, the relevance of this to treatment decisions may be limited. However, this level of risk from single genetic risk factors is seen for a range of complex diseases including many cancers and type II diabetes where large numbers of such risk factors have now emerged (48). Additive risks or interactions between risk factors may still determine overall risk. It is already clear that factors such as age and gender are also predictors of risk for some forms of idiosyncratic DILI. It may be possible to develop algorithms to assess individual risk of DILI in the future and these may include genotype for polymorphisms relating to drug disposition.

## ABBREVIATIONS

DILI: Drug-induced liver injury
GST: Glutathione S-transferase
GWAS: Genome-wide association study
HLA: Human leukocyte antigen
NAPQI: N-acetyl-p-benzoquinoneimine
NAT2: N-acetyltransferase 2
SNP: Single nucleotide polymorphism
UGT: UDP-glucuronosyltransferases
